# T-cell exhaustion due to BiTE therapy in multiple myeloma: mitigating infectious risks through treatment-free intervals

**DOI:** 10.3389/fimmu.2026.1705345

**Published:** 2026-01-21

**Authors:** Erin A. Dean

**Affiliations:** Hematopoietic Stem Cell Transplantation and Cellular Therapy Program, Division of Hematology/Oncology, Department of Medicine, University of California, Irvine, Orange, CA, United States

**Keywords:** bispecific T-cell engager therapy, BiTE therapy, CAR T-cell therapy, infections, multiple myeloma, T-cell exhaustion, treatment-free intervals

## Abstract

Multiple Myeloma (MM), a highly treatable but thus far incurable hematologic malignancy, has required continuous monitoring and treatment throughout a patient’s lifetime. Newer classes of therapies, such as chimeric antigen receptor (CAR) T-cell therapy which constitutes of a one-time infusion, have challenged the continuous treatment paradigm. Compared to previously known standard therapies, CAR T-cell therapy along with bispecific T-cell engager (BiTE) therapy have been observed to induce deeper responses that tend to be durable in responders. However, the degree of immunosuppression and infection noted especially with BiTE therapies, currently approved for ongoing administration until progression of disease, has given rise to the idea of incorporating treatment-free intervals. This review describes T-cell exhaustion as a driver for immunosuppression and infection in patients with MM receiving BiTE therapy and discusses potential ways to overcome it to improve management of patients.

## Introduction

Multiple Myeloma (MM) is a hematologic malignancy affecting the plasma cells in the patient’s immune system. Surface proteins on B-cells have been explored as potential treatment targets leading to the development of a few targeted therapies.

The receptor and ectoenzyme CD38, first identified as T10, is seen on cells of both hematopoietic and non-hematopoietic origin (1). Its high expression and uniform distribution on MM cells have made it an effective target for therapeutic anti-CD38 monoclonal antibodies (1). The mechanism of action for the first anti-CD38 therapy, daratumumab, is through complement-dependent cytotoxicity (CDC), antibody-dependent cellular cytotoxicity (ADCC), antibody-dependent cellular phagocytosis (ADCP), programmed cell death via Fcγ receptors crosslinking, and modulation of CD38 enzymatic function leading to depletion of CD38 immunosuppressive cells (1, 2). Isatuximab, the other commercially available anti-CD38 therapy, exerts its effects through CDC, ADCC, ADCP, proapoptotic function without crosslinking, and inhibition of CD38 ectoenzyme functioning (1). The difference in the observed mechanisms of action between the two anti-CD38 monoclonal antibodies can be explained by targeting of distinct epitopes on CD38 (1).

B-cell maturation antigen (BCMA), also known as TNFRSF17 or CD269, is a receptor found mainly on normal and malignant mature B lymphocytes that has been used as another target in MM (3). It functions via B-cell-activating factor (BAFF) and a proliferation inducing ligand (APRIL) which serve as ligands for the BCMA receptor that trigger differentiation of B cells and the production of antibodies (3). It is unique in that surface-bound BCMA can be cleaved off by γ-secretase to become soluble in the bone marrow supernatant and peripheral blood (4).

Finally, G protein–coupled receptor class C group 5 member D (GPRC5D) has higher expression on MM cells over other hematologic malignant cells, which has made it an effective therapeutic target in MM (5). GPRC5D differs from CD38 and BCMA, in that its expression is mainly in cells with plasma cell phenotype with limited to no expression in other cells in the immune cell compartment (5). GPRC5D is also unique in that it has epithelial distribution within the hair follicles, eccrine glands, skin, and tongue, which can lead to on-target off-tumor activity (5). The expression of GPRC5D and BCMA on cells is known to be independent, which confers the ability to employ therapy towards one target upon loss of the other (4). Even though GPRC5D’s signaling mechanism and function have not yet been elucidated, its structure and position within the cell membrane have been determined. GPRC5D has been shown to be 7-fold-embedded in the cellular membrane with a short extracellular domain establishing it as likely to remain cell-bound instead of shed (5). This differentiates it from BCMA which can become soluble, potentially reducing BCMA’s efficacy as a therapeutic target.

Bispecific T-cell engager (BiTE) therapy serves to bring T cells in proximity to the malignant B cells via surface proteins. There are four commercial BiTE products available for the treatment of relapsed/refractory (R/R) MM. Elranatamab, teclistamab, and linvoseltamab target CD3 on T cells and BCMA on MM cells and were approved based on results of the MagnetisMM-3, MajesTEC-1, and LINKER-MM1 trials, respectively; while talquetamab targets CD3 on T cells and GPRC5D on MM cells and was approved based on results of the MonumenTAL-1 trial (6).

Following the positive results of the pivotal trials, the recommended dosing for the BiTE therapies was either weekly or biweekly. This work summarizes the infectious risks associated with continuous use of BiTE therapy as per trial scheduling in the management of patients with R/R MM and presents evidence behind employing treatment-free intervals as a potential risk mitigation strategy.

## Immunosuppression and risk for infection in MM

Patients with either newly diagnosed or R/R MM, independent of receipt of treatment, experience humoral immunodeficiency with marked hypogammaglobulinemia leading to increased risk of infection compared to healthy adults (3). Hypogammaglobulinemia is typically defined as polyclonal immunoglobulin (Ig) G levels < 400 mg/dL (6). The suppression of normal B cells has been linked in experiments to secretion of inhibitors by tumor cells (7, 8). *In vitro* studies comparing peripheral blood samples of healthy individuals and patients with MM confirmed lower IgG levels in MM due to the presence of B-cell-suppressive phagocytic mononuclear cells in the circulating lymphocyte population (9). Investigations in mice models carrying plasmacytomas showed indirect suppression of normal B cells because of negative effects of tumor cells on the regulatory function of splenic and peritoneal macrophages (10). In addition to the role of inhibitors and host suppressor cells in hypogammaglobulinemia, after its discovery, soluble BCMA was also shown to play a part. Studies in mice and humans demonstrated that increased levels of soluble BCMA from MM cells result in sequestration of BAFF reducing surface-bound BCMA-BAFF interactions thus prohibiting the stimulation of B-cells and secretion of polyclonal antibodies (3). This mechanism was proved to be independent of monoclonal protein production by tumor cells, as a result of which, it has been observed in both secretory and non-secretory MM (3). As the function and role of other receptors is unraveled, more mechanisms of hypogammaglobulinemia development may be elucidated in the future.

Hypogammaglobulinemia and infections may present themselves or be worsened by the initiation of some targeted therapies for MM. Retrospective analyses of serum IgG levels in patients receiving anti-CD38 monoclonal antibody therapy have revealed the chance of hypogammaglobulinemia and infectious complications. In a retrospective study of hypogammaglobulinemia in the setting of daratumumab, 614 patients with MM were identified and their pre- to post-therapy mean IgG levels (adjusted by monoclonal protein subtraction for IgG type of MM) showed a significant reduction from 532 to 350 mg/dL (11). A greater percentage of patients, 89.6% from 69.3%, had low IgG levels post-daratumumab, and in those with preceding low IgG levels, the rates of moderate or severe hypogammaglobulinemia increased to 47.4% from 30.8% and 34.7% from 12.4%, respectively (11). Low IgG levels were associated with increased risk of infection and mortality (11). On the other hand, in a retrospective analysis of monotherapy isatuximab in 3 studies, therapy was not found to cause low IgG levels (12). When hypogammaglobulinemia was present, isatuximab in combination with either pomalidomide plus dexamethasone per the ICARIA-MM study or carfilzomib plus dexamethasone per the IKEMIA study was associated with increased risk of any grade infection but not Grade 3 or higher infection compared to the doublets without isatuximab (12).

The prevalence of hypogammaglobulinemia and infection associated with BiTE therapy for R/R MM were described in detail in the pivotal trials leading to their approval. These data have been of interest given the use of this new class of drugs in a heavily pre-treated patient population that already carries the intrinsic infectious risks occurring with persistence of MM.

On the MagnetisMM-3 trial, 123 patients received elranatamab once-weekly after step-up dosing; after 6 cycles of therapy, responders were switched to biweekly dosing (13). During treatment, hypogammaglobulinemia (IgG < 400 mg/dL) was noted at least one time in 75.5% of cases with 43.1% of patients requiring immunoglobulin replacement (13). Neutropenia (any grade, Grade ≥ 3) occurred in 48.8%, 48.8% of cases; infection (any grade, ≥ Grade 3) in 69.9%, 39.8%. For patients switched to biweekly dosing (n= 50), the incidence of neutropenia (any grade, Grade ≥ 3) decreased from 34.5% to 27.6%, 32.8% to 25.9%; while infections of any grade decreased from 53.4% to 48.3% but Grade ≥ 3 increased slightly from 20.7% to 22.4% (13). Out of the 55 deaths on the trial, 8 were due to infection with 3 (adenoviral hepatitis; adenovirus infection with pneumonia; pseudomonal pneumonia) attributed to the therapy by the investigators (13).

On the MajesTEC-1 trial, 165 patients were enrolled and treated with once-weekly teclistamab after step-up priming dosing (14). Hypogammaglobulinemia (IgG < 500 mg/dL) occurred in 74.5% of patients with 52.8% of them receiving immunoglobulin replacement (14). Neutropenia (any grade, Grade ≥ 3) was recorded in 70.9%, 64.2% in cases; infections (any grade, Grade ≥ 3) in 76.4%, 44.8% (14). For neutropenia, 55.2% of all patients received granulocyte colony-stimulating factor therapy (14). Out of the 68 deaths on the study, 19 were due to infection with 2 cases of COVID-19, 1 case of progressive multifocal leukoencephalopathy, and 1 case of streptococcal pneumonia attributed to the drug (14).

On the LINKER-MM1 trial, 117 patients received 200 mg linvoseltamab once-weekly for 14 weeks, then every other week and monthly after week 24 if they achieved at least very good partial response (VGPR) (15). Neutropenia (any grade, Grade ≥ 3) occurred in 42.7%, 41.9% of patients and infection (any grade, Grade ≥ 3) in 74.4%, 35.9% of patients (15). Out of the 35 deaths on the study, 3 infectious deaths due to *Pneumocystis jirovecii* pneumonia, progressive multifocal leukoencephalopathy, and pseudomonal sepsis were attributed to the drug (15).

On the MonumenTAL-1 trial, 232 patients received talquetamab once-weekly (405 μg/kg) or every other week (800 μg/kg) with or without step-up dosing as per assignment (16). Hypogammaglobulinemia (IgG < 500 mg/dL) was documented in 87% of patients who received the weekly dose and 71% who received the every other week dose (16). Neutropenia (any grade, Grade ≥ 3) was measured in 67%, 60% of patients dosed weekly; and in 36%, 32% of patients doses every other week (16). Infections (any grade, Grade ≥ 3) occurred in 47%, 7% of patients doses weekly; and in 34%, 7% in patients dosed every other week (16). One of the 3 deaths on the study was due to infection (sepsis) but was not considered secondary to talquetamab (16).

**Table 1** summarizes the immune complications of the BiTE therapies along with reported interventions.

**Table 1:** Immunocompromise and Interventions during BiTE Therapy on Trial in R/R MM.

BiTE Therapy, Trial	Elranatamab, MagnetisMM-3 trial (n= 123), %	Teclistamab, MajesTEC-1 trial (n= 165), %	Talquetamab, MonumenTAL-1 trial (n= 232), %	Linvoseltamab, LINKER-MM1 trial (n= 117), %
Adverse event				
Hypogammaglobulinemia	75.5	74.5	87 (weekly dose) 71 (every other week dose)	Not reported
Neutropenia (any grade, Grade ≥3)	48.8, 48.8 n= 50: 34.5, 32.8 (weekly)-> 27.6, 25.9 (every other week)	70.9, 64.2	67, 60 (weekly dose) 36, 32 (every other week dose)	42.7, 41.9 (at 200 mg)
Infection (any grade, Grade ≥3)	69.3, 39.8 n= 50: 53.4, 20.7 (weekly)-> 48.3, 22.4 (every other week)	76.4, 44.8	47, 7 (weekly dose) 34, 7 (every other week dose)	74.4, 35.9 (at 200 mg)
Intervention				
Immunoglobulin	43.1	52.8	Not reported	Not reported
Granulocyte colony-stimulating factor therapy	Not reported	55.2	Not reported	Not reported
Prophylactic antimicrobials antiviral anti-*Pneumocystis jirovecii* antifungal antibacterial	87 49.6 11.4 5.7	Not reported	Not reported	Not reported started mid-study

Hypogammaglobulinemia was defined as IgG < 400 mg/dL on MagnetisMM-3 and IgG < 500 mg/dL on MajesTEC-1, MonumenTAL-1.

## T-cell exhaustion and strategies to overcome immunosuppression

Balancing efficacy with inherent risks of infection is necessary for achieving good clinical outcomes with BiTE therapy in R/R MM. In addition to starting prophylactic antimicrobials and as needed administration of replacement immunoglobulin and supplemental granulocyte colony-stimulating factor therapy as per consensus recommendations of MM experts (17), alternate dosing schedules to indefinite therapy have also been explored to curb immunosuppression while preserving response. The main rationale behind spacing out therapy has been based on increased understanding of how T-cell exhaustion observed with indefinite therapy can precipitate immunosuppression (18).

T-cell exhaustion can occur in the context of many chronic infections and cancer (19). Exhaustion is due to continued antigen stimulation or tonic receptor signaling (19). It is characterized by poor T cell effector function, the expression of inhibitory receptors, and an altered transcriptional state, which in combination prohibit the body’s control of infections or cancer (20). The phenomenon has been studied in chronic infections, such as chronic lymphocytic choriomeningitis virus, simian immunodeficiency virus, hepatitis C virus, and herpes simplex virus (21). In mice models with lymphocytic choriomeningitis virus infection, T cell receptor-induced transcription factors, IRF4, BATF, and NFATc1 led to CD8(+) T cells dysfunction by causing overexpression of the inhibitory receptor PD-1, decreasing TCF1 expression thus blocking T cell differentiation, and weakening cellular metabolism (22). In many solid cancers, such as melanoma and lung cancer, the ligand PDL1 (also called B7-H1 and CD274) is overexpressed by tumor cells which in turn prohibits T-cell mediated anti-tumor activity (23). In a similar way, in some B cell lymphomas, the ligand PDL2 (also called B7-DC and CD273) is upregulated on malignant cells impairing T cell responses (23). In a mouse model study of a tamoxifen-induced liver cancer, tumor-specific CD8(+) T cells displayed abnormal phenotypic, functional, and transcriptional changes early during tumorigenesis that were reversible only initially through treatment (24). The T-cell exhaustion observed during the first stages of tumorigenesis differed from the process in chronic infections (24). While T-cell exhaustion is the body’s natural moderation mechanism against extreme immune system damage, it can prevent the clearance of tumor cells and viruses (22).

T-cell exhaustion can occur in the setting of certain therapies, including chimeric antigen receptor (CAR) T-cell therapy and BiTE therapy (19). For example, in earlier mice models of pleural mesothelioma, CAR T cells became exhausted following PD-1 overexpression and abnormal expression of other effector molecules, such as IFN-γ and TNF-α (25). In *in vivo* tumor models, exhaustion of CAR T cells was associated with deficiency of the canonical AP-1 factor c-Jun, which was overcome by manufacturing of c-Jun overexpressing CAR T cells that displayed improved expansion, functioning, anti-tumor activity, and decreased terminal differentiation (26). CAR T-cell therapy exhaustion has been reported in non-responders with chronic lymphocytic leukemia and B-cell lymphoma (27). In patients with R/R MM receiving anti-BCMA CAR T-cell therapy, early relapse of disease has been associated with exhausted CD4(+) CAR T cells (28).

T-cell exhaustion has been identified with BiTE therapy and anti-CD38 monoclonal antibodies as well. A study of the anti-CD19 x anti-CD3 BiTE, blinatumomab, in patients with R/R B-cell acute lymphoblastic leukemia noted a diminishing T cell function during the 28-day continuous therapy infusion due to decreasing cytokine production, as well as decreasing T cell proliferation and cytotoxicity (19). The same loss of T cell function has been observed with anti-BCMA and anti-GPRC5D BiTE therapies (29). The two anti-CD38 therapies, daratumumab and isatuximab, have also been observed to cause decrease in regulatory T cells (30).

As T-cell exhaustion is becoming better recognized as a limiting factor associated with newer treatments such as CAR T-cell therapy and BiTE therapy, processes are being investigated to overcome or prevent it. In CAR T-cell therapy, blocking the PD-1/PD-L1 axis, increasing expression of c-Jun, or deletion of the transcription factors TOX, TOX2, or other NR4A family transcription factors by CRISPR may be ways to restore T cells (31). The main downside of these T-cell exhaustion reversal methods is their inability to also restore the epigenetics of normal functioning T cells (31). An *in vitro* model devised to integrate rest periods to allow reinvigoration of CAR T-cell therapy via downregulation of the CAR protein using a drug-regulatable system or treatment with the drug dasatinib was able to achieve epigenetic recalibration in addition to recovery of the T cells’ memory-like phenotype, original transcriptional status, and anti-tumor ability (31). Investigators demonstrated prevention of T-cell exhaustion by inhibiting CAR signaling before T-cell exhaustion was observed or reversal of T-cell exhaustion after it had occurred and CAR signaling was paused (31).

In BiTE therapy, one of the main techniques proposed to minimize T-cell exhaustion rests on the same idea of reducing chronic antigen stimulation, however, by limiting the exposure to BiTE therapy through treatment-free intervals (18). It is unknown if upon restarting immunotherapy, however, resistance might develop like with targeted small molecule therapies (32). In an experiment involving an *in vitro* model of an anti-CD19 x anti-CD3 BiTE called AMG 562, continuous exposure of T cells to the drug led to decrease in T cell function as measured by mean specific lysis from 88.4% to 8.6% between day 7 and day 28 of therapy (18). Comparing continuous administration of AMG 562 day 1–14 to day 1–6 only (with a treatment-free interval day 7-14), the day 14 specific lysis was 34.9% versus 93.4%; comparing the samples after continuing AMG 562 uninterrupted until day 28 versus day 15-20 (with a second treatment-free interval day 21-28), the day 28 specific lysis became 8.6% versus 58.7% (19). Taking all results of the study in consideration, the investigators concluded that treatment-free intervals contributed to restoration of T cell function and transcriptional reprogramming; additionally, *in vivo*, it enhanced T cell expansion and tumor killing (19).

**Figure 1:**
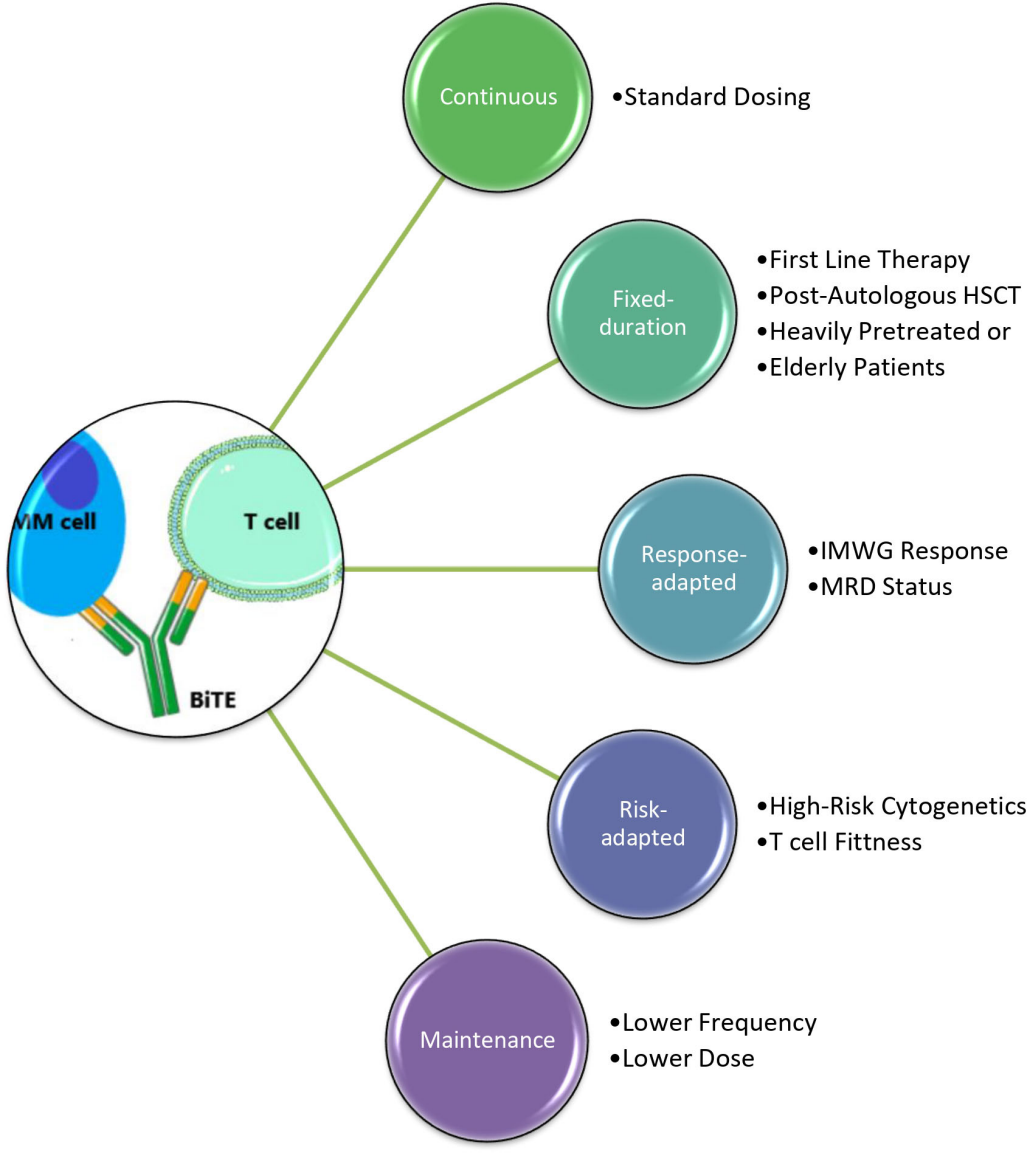
Various novel approaches of BiTE therapy administration for MM. BiTE therapy works by bringing T cells close to MM cells. Continuous BiTE administration is initially either weekly or biweekly per standard dosing and continues until progression of disease. Alternate fixed-duration, risk-adapted, and response-adapted schedules are being devised with significant considerations listed here. Maintenance therapy may be used after limited duration therapy. *Image adapted from Servier Medical Art (**https://smart.servier.com/**), licensed under CC BY 4.0 (**https://creativecommons.org/licenses/by/4.0/**).*.

These studies imply that, in theory, treatment-free intervals during BiTE therapy may mitigate the risk of immunosuppression, infection, and reduced tumor-killing due to T-cell exhaustion. In cases where therapy was held or discontinued because of adverse events, most patients retained their response and the durable responses were attributed to the initial cellular and humoral immune response against multiple tumor antigens, known as antigen spreading (29). **Figure 1** lists possible new BiTE therapy administration approaches (29).

Fixed-duration treatment with BiTE therapy is supported by data from several studies and is currently under investigation. In MajesTEC-1, teclistamab was on hold for 23 of 104 responding patients for a median duration of 4.7 (range 3.3–21.5) months (33). In LimiTec, a phase II single-arm study, patients with MM status post at least 3 prior lines of therapy, teclistamab discontinuation after 6–9 months and achievement of VGPR resulted in median failure free survival of 73% at 12 months (34). If progression of disease occurred at < 6 months, BCMA loss was observed, which the authors did not suspect was due to discontinuation of therapy (34). The EMN37 fitfix study is a planned prospective clinical trial to evaluate progression free survival at 18 months for frail patients per the International Myeloma Working Group (IMWG) Frailty Index with newly diagnosed MM who will be randomized to fixed-duration therapy (18 cycles) with either teclistamab-daratumumab or talquetamab-daratumumab treatment (35). These studies and individual cases focus on frail or heavily pretreated patients with MM.

Response-adapted length of BiTE therapy after achievement of VGPR or CR per IMWG criteria and/or measurable residual disease (MRD) negativity is also of great interest since after only 1–4 months of anti-BCMA or anti-GPRC5D BiTE therapy patients can achieve MRD negativity and since most patients in CR test negative for MRD (29). This approach can allow transitioning from treatment to maintenance therapy and thus far has been best studied in this setting in patients with R/R MM. In MajesTEC-1, patients with durable responses were allowed to switch from weekly to every other week dosing; they maintained deep responses at 69% for ≥ 2 years and had lower infection rate of 15.6% versus 33.3% at 1 year (36). In MagnetisMM-3, 80% of patients who switched from weekly to biweekly elranatamab retained response 6 months later (13). In LINKER-MM1, patients in VGPR after 24 weeks went on to once monthly dosing from biweekly with sustained response and lower infectious rates (15). In MonumenTAL-1, after 3 months of talquetamab, patients were allowed to switch to less frequent/lower potency dose and responses were retained (37).

Evaluation of risk-adapted strategies for shortening the length of BiTE therapy requires further investigation via dedicated prospective trials. Anti-BCMA and anti-GPRC5D BiTEs have resulted in similar response rates and PFS among patients with standard-risk cytogenetics and high-risk cytogenetics [del(17p), t(4;14), and/or t(14;16)] managed for R/R MM (29). Changes in T cell phenotype and fitness during BiTE therapy for R/R MM have been associated with clinical outcomes and thus may be able to guide treatment duration (29).

In the future, exploring the fixed-duration, response-adapted, and risk-adapted approaches for determining the duration of BiTE therapy when used as part of first line treatment in fit patients will also be important for minimization of toxicity.

In an Excel-based longitudinal model, extended dosing intervals of the 3 commercial anti-BCMA BiTE therapies were shown to reduce the patients’ time in contact with the healthcare system, also known as time toxicity (38). After initial weekly dosing, extended dosing was then every other week for linvoseltamab starting week 16, for elranatamab upon reaching at least partial response at ≥ 6 months, and for teclistamab upon reaching complete response at or after ≥ 6 months (38). Over 2 years, patients receiving linvolseltamab were found to have the lowest number of contact days with the biggest reduction from 59 to 39 days, but patients receiving either of the three BiTE therapies all had fewer number of contact days as a result of transitioning to an extended dosing schedule (38).

In addition to improved quality of life, reducing the frequency of treatments also has the potential to lower healthcare costs (29).

## Conclusion

Continuous BiTE exposure can result in T-cell exhaustion with subsequent immunosuppression. In addition to providing infectious prophylaxis and supportive care measures, treatment-free intervals may improve T cell functioning. Future research in the field should focus on evaluating the safety and efficacy of non-continuous administration of BiTE therapy for R/R MM. Administering BiTE therapy with pre-determined treatment-free intervals or as a fixed-duration course based on disease risk or response can serve to mitigate therapy-related infectious risks while maintaining efficacy.
